# Symmetry: Modeling the Effects of Masking Noise, Axial Cueing and Salience

**DOI:** 10.1371/journal.pone.0009840

**Published:** 2010-04-06

**Authors:** Chien-Chung Chen, Christopher W. Tyler

**Affiliations:** 1 Department of Psychology, National Taiwan University, Taipei, Taiwan; 2 Center for Neurobiology and Cognitive Science, National Taiwan University, Taipei, Taiwan; 3 The Smith-Kettlewell Eye Research Institute, San Francisco, California, United States of America; Rutgers University, United States of America

## Abstract

Symmetry detection is an interesting probe of pattern processing because it requires the matching of novel patterns without the benefit of prior recognition. However, there is evidence that prior knowledge of the axis location plays an important role in symmetry detection. We investigated how the prior information about the symmetry axis affects symmetry detection under noise-masking conditions. The target stimuli were random-dot displays structured to be symmetric about vertical, horizontal, or diagonal axes and viewed through eight apertures (1.2° diameter) evenly distributed around a 6° diameter circle. The information about axis orientation was manipulated by (1) cueing of axis orientation before the trial and (2) varying axis salience by including or excluding the axis region within the noise apertures. The percentage of correct detection of the symmetry was measured at for a range of both target and masking noise densities. The threshold vs. noise density function was flat at low noise density and increased with a slope of 0.75–0.8 beyond a critical density. Axis cueing reduced the target threshold 2–4fold at all noise densities while axis salience had an effect only at high noise density. Our results are inconsistent with an ideal observer or signal-to-noise account of symmetry detection but can be explained by a multiple-channel model is which the response in each channel is the ratio between the nonlinear transform of the responses of sets of early symmetry detectors and the sum of external and intrinsic sources of noise.

## Introduction

One of the major functions of the visual system is to identify and localize objects in a visual scene. To achieve this, we can assume that the visual system is likely to have developed means of utilizing many kinds of useful information. Mirror symmetry is one of the important image features, and is present in a large proportion of the objects that we encounter. In the wild, for instance, many relevant aspects of the environment, such as potential predators, food sources or mates, tend to have mirror symmetry while the background elements, such as rocks, water, trees, and hillsides, are largely non-symmetric [1]. Hence, the ability to extract symmetry information effectively should facilitate the identification of relevant objects in a complex scene [2], [3], [4]. Thus, it is not surprising that mirror symmetry detection is an ability that has been routinely demonstrated in mammals [5], [6], [7] and insects [8] and is an effortless or easy task for humans [9], [10] as a mere 50 ms presentation is usually sufficient for a human observer to tell a mirror symmetric stimulus from noise [9], [11], [12], [13].

While detecting mirror symmetry is easy for the human vision system [14] it is actually a complicated process from the computational point of view. By definition, a visual stimulus is mirror symmetric if some part of this stimulus is a reflection of another part about a certain axis. It is difficult to decide whether an image has two or more parts that are reflections of each other unless the location and orientation of the symmetry axis is specified; while one cannot determine the symmetric axis location unless two parts of the image are recognized as reflections of each other. Hence, the question of how the human visual system performs the novel pattern-recognition task required to resolve the symmetry is a chicken-and-egg problem.

Currently, the spatial filtering approach is popular framework for understanding symmetry perception [15]–[23]. While there is a considerable variation in detail, spatial filtering models for symmetry perception share many features. First, the input stage is modeled as a band of linear filters whose sensitivity profiles contain excitatory and inhibitory regions. There are data showing that these filters may be oriented [15], [20]. In some versions of the model, filters with different phase selectivity are required [19]–[22]. These filters operate on the input images. If an input image is symmetric, the filtered image would contain features at or across the symmetry axis that can be picked up by a second-order filter at the orientation of the symmetry axis [17], [22] or by a simple mathematical operator operating orthogonal to the symmetry axis [15], [16], [19]–[21]. For these models to work, however, one has to make an assumption about the location and orientation of the symmetry axis, on which all the operations on the filtered image depend. However, mirror symmetry can occur at any orientation in a nature scene. Thus, while current spatial filtering models perform well to explain the data from experiments with a known symmetry axis orientation, their generality is limited as they have not addressed the situation whether the symmetry axis orientation is unknown to the observer.

The purpose of our study is then to understand the effect of uncertainty about axis orientation in the framework of Signal Detection Theory [24]. We measured symmetry detection thresholds for target dot patterns with one of four possible symmetry axis orientations. The target patterns were embedded in different amounts of noise. Such manipulations allow us to characterize the functional relationship between the input stimuli and the internal response of the visual system and decision process. Lu & Dosher [25] developed a similar experimental paradigm in the domain of contrast detection. The advantages of such a paradigm were recently reviewed by Lu & Dosher [26].

Here, we consider two possible hypotheses as to how the visual system determines the axis orientation for the detection of symmetry. The first hypothesis assumes that a higher-order symmetric detector receives the responses from lower-order mechanisms that are each sensitive around a symmetry axis of a particular orientation. When the axis orientation is unknown to the observer, according to the uncertainty theory [27], [28], a higher-order detector needs to monitor the output of all lower-order symmetry detectors at all possible axis orientations and in turn makes the decision based on the maximum response among all lower-order mechanisms. On the other hand, if the axis orientation is known to the observer, the system needs to monitor only the lower-order mechanism whose orientation selectivity matches that the symmetry axis. The ideal observer would thus switch strategy to match the known stimulus conditions. Thus, symmetry detection thresholds measured at different numbers of possible symmetry axes can be predicted by the relative levels of uncertainty in the system.

The second hypothesis suggests that the visual system may simply analyze the spatial relationships among individual image elements and determine an image to be symmetric if a sufficient proportion of the spatial locations of image elements support it. That is, symmetry detection would be based solely on the signal-to-noise ratio or “weight-of-evidence” in the image [29], [30]. In the context of our experiment, suppose that the observer has the knowledge that an image, if it is symmetric, had, say, vertical symmetry axis through the fixation point. The visual system then analyzes how many image elements have a horizontal correspondence relative to that axis location and determine that the image is symmetric if the proportion of elements with such horizontal correspondence reaches a baseline criterion level. On the other hand, if the symmetry axis orientation is unknown to the observer, the visual system would need to analyze the correspondence of an image element over many different orientations. In this case, since the image, and in turn, the number of elements supporting a symmetry judgment, is the same while the spatial relationships needing to be examined increased, it is a more difficult task to determine whether the image is symmetric. Thus, an increase in the number of possible axis orientations increases the number of symmetric dot pairs in the image needed for it to be judged as symmetric. That is, the lack of knowledge of axis orientation effectively reduces the sensitivity of the visual system to the symmetry information in the image.

In addition to the axis orientation, we also need to consider the issue of salience of symmetry axis, which is relevant to how the location of symmetry axis is determined in some models. For instance, Rainville & Kingdom [20] used detectors with adjacent and aligned filters of opposite polarities to process the input images. Such detector would produce a zero response at the symmetry axis of a symmetric image and a non-zero response elsewhere. The models proposed by Gurnsey et al. [17], Osorio [6] and Scognamillo et al. [22] also used the idiosyncratic filter responses at or near the symmetry axis to determine the location of the axis. This requirement, however, contradicts the result that a human observer is able to perceive symmetry based on image elements far away from the symmetry axis [9], [31]. Indeed, Tyler & Hardage [1] found no diminution of the detectability of symmetry even for pattern regions separated by 60 degrees of visual angle (as long as the element size was scaled with eccentricity). We will define the degree to which the symmetry axis is present in the pattern as the “axial salience” of the pattern symmetry. The further the pattern elements are from the geometric location of the symmetry axis, the less salient that axis is considered to be. To examine the effect of axial salience, we had our observers view the stimuli through a mask of six apertures. The apertures were arranged to control whether the part of the image around the symmetry axis was visible to the observers. With this manipulation, we can quantitatively estimate the effect of axial salience on symmetry detection.

## Methods

### Ethics statement

The use of human participants was approved by the IRB of National Taiwan University Hospital and followed the guideline of Helsinki Declaration. The written informed consent was obtained from each participant.

### Apparatus

The stimuli were presented on a ViewSonic VA902 17″ LCD monitor controlled by an HP D325MT computer with an ATI Radeon 9800PRO graphics card. The spatial resolution was 1280 (H) × 1024 (V). At the viewing distance of 83.2 cm, a pixel subtended 1′ (H) × 1′ (V). The temporal refresh rate of the monitor was 60 Hz (non-interlaced). The gamma function of the monitor was calibrated with a LightMouse photometer [32], and this information was used to compute linear 8-bit color look–up table. The accuracy of the look-up tables was verified by an international Light RPS-380 spectroradiometer. The experimental control software was written in MATLAB with the Psychophysics Toolbox [33]. The display had mean luminance at 15 cd/m^2^ and chromaticity of (0.33, 0.33) in CIE 1931xycoordinates.

### Stimuli

In our experiment, the information about the symmetry axis was manipulated in two ways. The information about the axis was varied by (1) cueing: whether there was a cue indicating the axis orientation before a trial; and (2) axial salience: whether the axis location fell within the apertures or between them.

**Figure 1:**
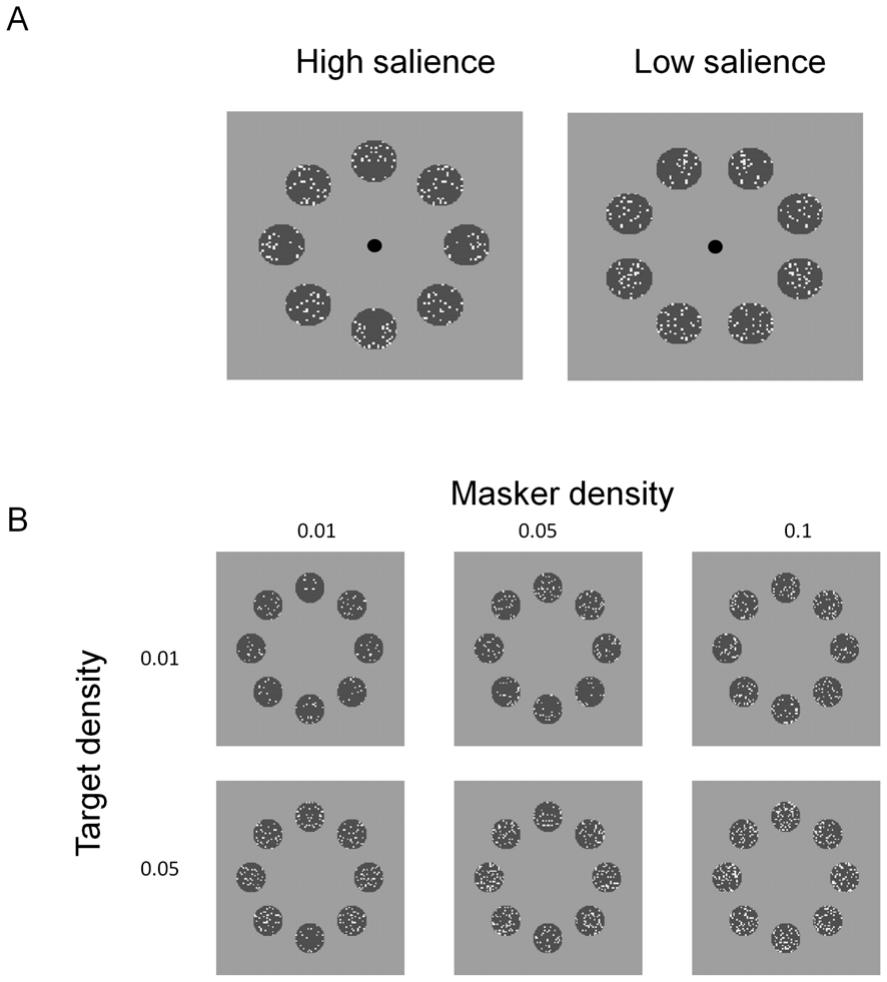
Examples of stimuli. A. The configuration consisted of an overall noise pattern with a single axis orientation visible through a mask of eight apertures The axial salience was controlled by the position of the apertures, located so as to either include or exclude the region around the axis. B. Examples of different combinations of target and masker density.

Figure 1 shows examples of the stimuli. The dot patterns consisted of white (30.1 cd/m^2^) pixels (4′×4′) randomly distributed on a black (0.2 cd/m^2^) background. The density of the random-dot mask varied from 0 to 10%. In the symmetric target, half of the displays structured to have symmetry about an axis whose orientation was either vertical, horizontal, or one of the two diagonals. That is, a pixel at position (x,y) of the symmetric image I has the property 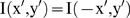
(1)where
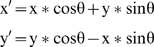
where θ denotes the four possible orientations of the symmetry axis with θ  = 0° for the vertical, θ  = 90° for the horizontal, and 45° and 135° for the two diagonal symmetry axes.

On each trial, the stimuli consisted of a random-dot mask superimposed on either a symmetric target or a non-symmetric random-dot control. The purpose of the random-dot control was to balance the local statistics in the image. The stimuli were spatially masked with a uniform gray field (15 cd/m^2^) with eight apertures (1.2° diameter) evenly distributed around a 6° diameter circle. In the high axial salience condition, the centers of the apertures were located from 0° to 315° in 45° steps from the horizontal axis to include the symmetry axis in diametrically-opposite pairs, regardless of which of the four orientations the axis took. In the low axial salience condition, the centers of the apertures were shifted clockwise by 22.5° to exclude the symmetry axis from all the apertures. In this configuration the blank region around each possible axis location was a minimum of 1.16°.

### Procedure

On each trial, observers determined whether a symmetric target or a non-symmetric control pattern was presented. The axis was randomly selected from one of the four orientations on each trial, but information about the axis was manipulated by (1) cueing and (2) axial salience. Thus, there were a total of 4 ( = 2×2) test conditions in the experiment. In the cue condition, a straight line with the same orientation and location as the symmetry axis flashed for 500 ms, followed by 15 ms of a uniform gray field, before the onset of the stimuli. In the non-cue condition, instead of the valid cue, a neutral cue of four lines that had the same orientations and location as the four possible symmetry axes was presented before the test stimuli. The test stimuli stayed on the screen until the observer made a response, after which the display was replaced by the uniform gray field. The salience and non-salience conditions were determined by the location of the apertures as discussed above.

The trials were blocked by test condition as well by the noise density, but axis orientation was randomized throughout each block. In each block, we used a constant stimulus paradigm to measure the psychometric functions of percentage correct responses for a range of 7–9 target densities in 0.15 log increments. The range of target densities depended on both test conditions and noise density and was determined by a pilot experiment (data not shown) in which one of the authors served as an observer. The sequence of target density and axis orientation within a block, or noise density and test conditions between blocks, were all randomized.

Four observers participated in this study. One observer (CC) was one of the authors of this paper while the other three were paid observers who were naïve to the purpose of the experiment. All observers had a corrected–to-normal (20/20) visual acuity. Observer PC left the study before making measurements with the low-density noise masks.

## Results

**Figure 2:**
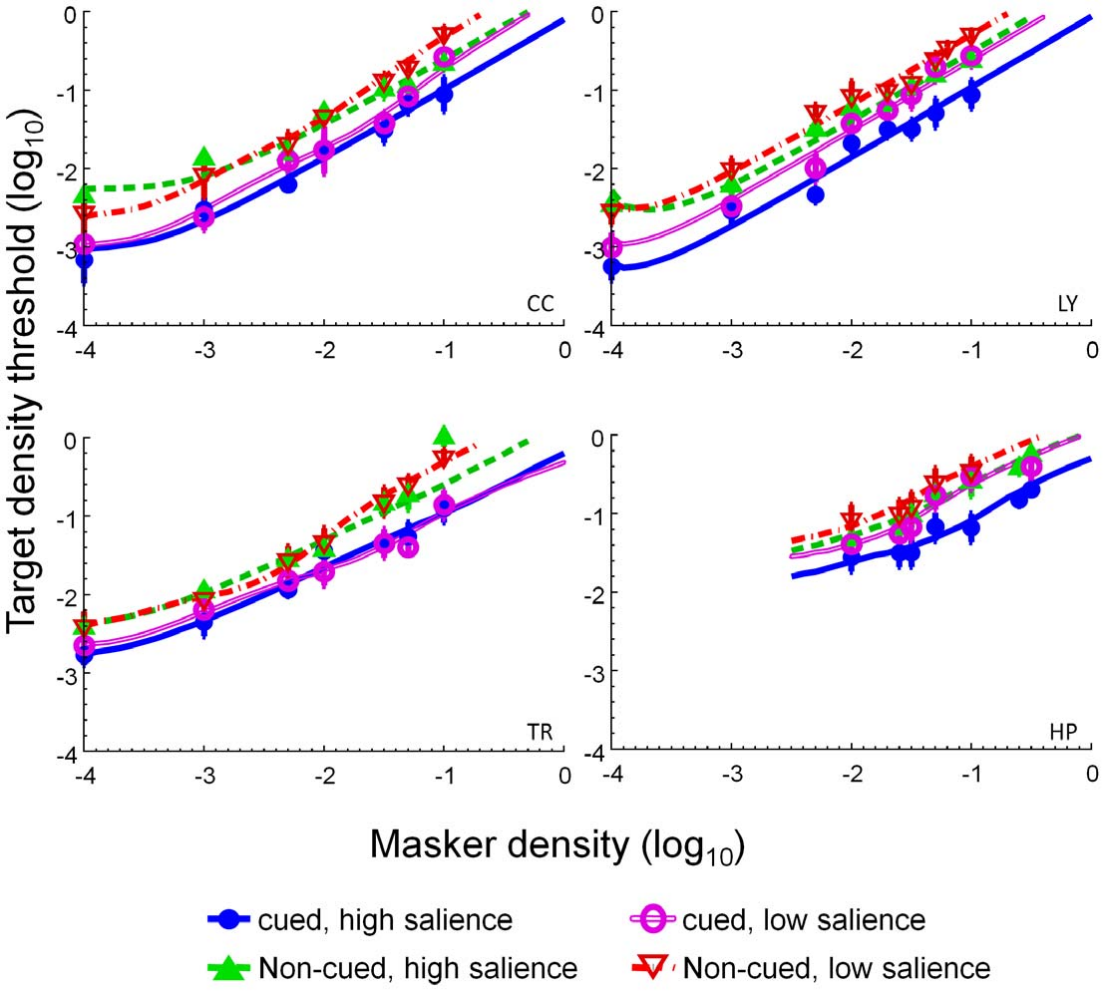
Target threshold vs. masker density functions. Each panel represents data from one observer. Blue denotes the TvD function for the cued high salience condition; magenta, the cued low salience condition; green, the non-cued high salience condition; and red, the non-cued low salience condition. The smooth curves are fits of the model discussed below. The error bars are the estimated standard error of measurement.

Figure 2 shows the target threshold vs. noise density (TvD) function for four conditions. Each panel in Figure 2 represents the TvD functions from one observer. Blue symbols denote the TvD function for the cued high axial salience condition; magenta symbols, the cued low axial salience condition; green symbols, the non-cued high axial salience condition; and red symbols, the non-cued low axial salience condition. The smooth curves are fits of the model discussed below.

For all conditions, the target density threshold increased with noise density. At medium to high noise densities, the slope of the increment function reached an average of about 0.77 in log-log coordinates for all conditions and observers, significantly less than a slope of 1 (t(15) = 6.19, p<0.001). The asymptotic slope of the TvD functions varied with axial salience. Averaged across observers and cue conditions, the TvD functions for the low salience conditions had a slope (0.86) significantly greater (t(7) = 2.38, p = 0.048) than that for the high salience conditions (0.70). Within the same salience condition, there is little difference is slope for TvD functions measured for different cueing conditions (t(7) = 0.65, p = 0.53). At the low noise densities, the slope of the increment function may be less because the density thresholds measured with no masking noise would be the same as those measured at noise densities between −3 and −4 log units (as predicted from the slope at the high noise densities).

**Figure 3:**
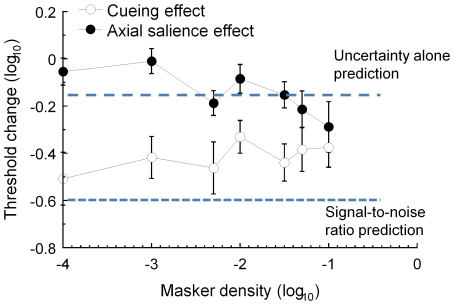
The average threshold change produced by cueing (open circles) and axial salience (closed circles) at different masker densities. The dashed and dotted blue lines indicate the predictions of the uncertainty model and the signal-to-noise, or weight-of-evidence, model respectively.

Wenderoth [34] studying symmetric stimuli with no external noise, reported that cueing the axis orientation facilitated symmetry detection. We now show that, regardless of the degree of axial salience, the axis cue produced a facilitative effect on symmetry detection. The open circles in Figure 3 denote the threshold difference between the cued and non-cued conditions, averaged across observers and salience conditions. The magnitude of the threshold reduction was from 0.3 to 0.6 log unit (or a two- to four-fold change) across observers. These large cueing effects were about the same for all noise densities.

The filled circles in Figure 3 denote the threshold difference between the high and the low axial salience conditions, averaged across observers and cueing conditions. In contrast to the cueing effects, there was little salience effect on symmetry threshold at low noise density (t(5) = 0.94, p = 0.19). The salience effect increased with noise density reaching up to ∼0.3 log unit, or a two-fold change, by cueing the axis location at the highest noise density.

## Discussion

The experiment was designed to directly compare two aspects of the knowledge about the symmetry axis on the detectability of symmetry as a function of masking noise density.

### Threshold effects

The axis orientation cue reduced the symmetry detection threshold 2–4 fold. This axial salience effect was pronounced at high but not low noise densities. The threshold reduction produced by the cue is inconsistent with what would be predicted by a simple signal-to-noise ratio or weight-of-evidence account of symmetry detection [29], [30]. In our experiment, there were four possible axial orientations. Hence, the observer needed to inspect the spatial relationships between dots over four possible orientations when the orientation of the symmetric axis was unknown. That is, compared with the non-cued condition, the observer needed to compare four times fewer dot relations in the cued conditions. As a result, we would expect a 4-fold improvement in threshold (denoted as dotted line in Figure 3) by the informative cue. This is, in general, an overestimation of the cue effect, which averaged 3-fold.

The effect of the cue, however, cannot be explained by uncertainty reduction alone. We assume that the observer's performance in both conditions is determined by the channel with the greatest response. Gaussian Max Uncertainty Theory [35] predicts that the signal intensity in the non-cued condition over four axes to be 1.7 times greater than that in the cued condition to maintain the same discriminability, or d'. Thus, the effect of the cue would be a 1.7 times, or 0.15 log unit, decrease in threshold. The dashed horizontal line in Figure 3 denotes this threshold reduction. Our results all showed a greater effect than the uncertainty model predicts. Hence, the cueing must be affecting more than just uncertainty in our experiment.

The axial salience effect was pronounced at high but not at low noise densities. Actually, when there was no external noise, the salience effect did not differ significantly from zero. Given that the gap between the neighboring apertures was at 1.16° at their closest, a lack of threshold difference between the high and low salience condition the observer is not using the information close to the symmetry axis for symmetry detection. Since models of symmetry based on the image property at or near the symmetry axis [17], [18], [20] would predict an advantage from the high axial salience in symmetry detection, our result suggests that such models are, at best, valid only under high-noise viewing conditions.

### Slope of increment threshold function

Our results showed that the increase of target density threshold with noise density had a slope of between 0.70 and 0.86 in log-log coordinates. This result is not consistent with the simple signal-to-noise-ratio [9] or weight-of-evidence [29], [30] accounts of symmetry detection. Van der Helm & Leeuwenberg [30], for example, suggested that symmetry detection is determined by the ratio of the number of symmetry pairs and the total number of image elements in the image. Hence, the number of symmetry pairs required for symmetry detection should increase proportional with the masker density. Scaled by the size of the image, the target density threshold should increase with the density of the noise masker with a slope of 1 on log-log coordinates. With a different approach, Barlow & Reeves [9], proposed that discriminability, d', of a random-dot symmetry pattern should be proportional to the difference between the number of symmetric pairs in the symmetric target+noise pattern, divided by the standard deviation of the number of the symmetric pairs in the noise pattern. This model would also predict that the target threshold, or the number of symmetry pairs that allows d' reaches a constant, increases with masker density with a slope asymptotic to 1.

**Figure 4:**
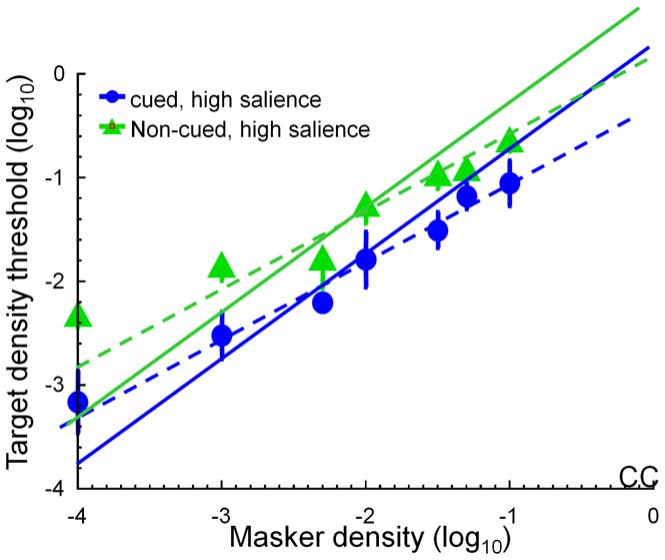
The slopes of the TvD functions. The solid lines have a slope of 1, the dashed lines, a slope of 0.75. The lines with unity slope tend to overestimate thresholds at high masker density and underestimate them at low masker density.

Our data do not fit with this picture. Figure 4 shows examples of our data and this prediction. At first glance, a line of slope of 1 (solid line) may give a visually passable fit across the data on this log-log plot, which spans four log units. More careful examination shows that this line overestimates the thresholds at high masker density while underestimates them at low density. On the other hand, lines of slope 0.75 (dashed lines) are much closer to the data points. Indeed, fitting the data in the masker density range between 0.001 and 0.1 with a line of slope 0.75 gives a sum-of square error of only 1/3 of that for the slope 1 fit. However, as shown in Figure 2, the slope of the TvD function depends on the test conditions. Hence, a line of any particular slope cannot account for all our data. Slope must be a free parameter in a well-fitting model of these data.

### Model

**Figure 5:**
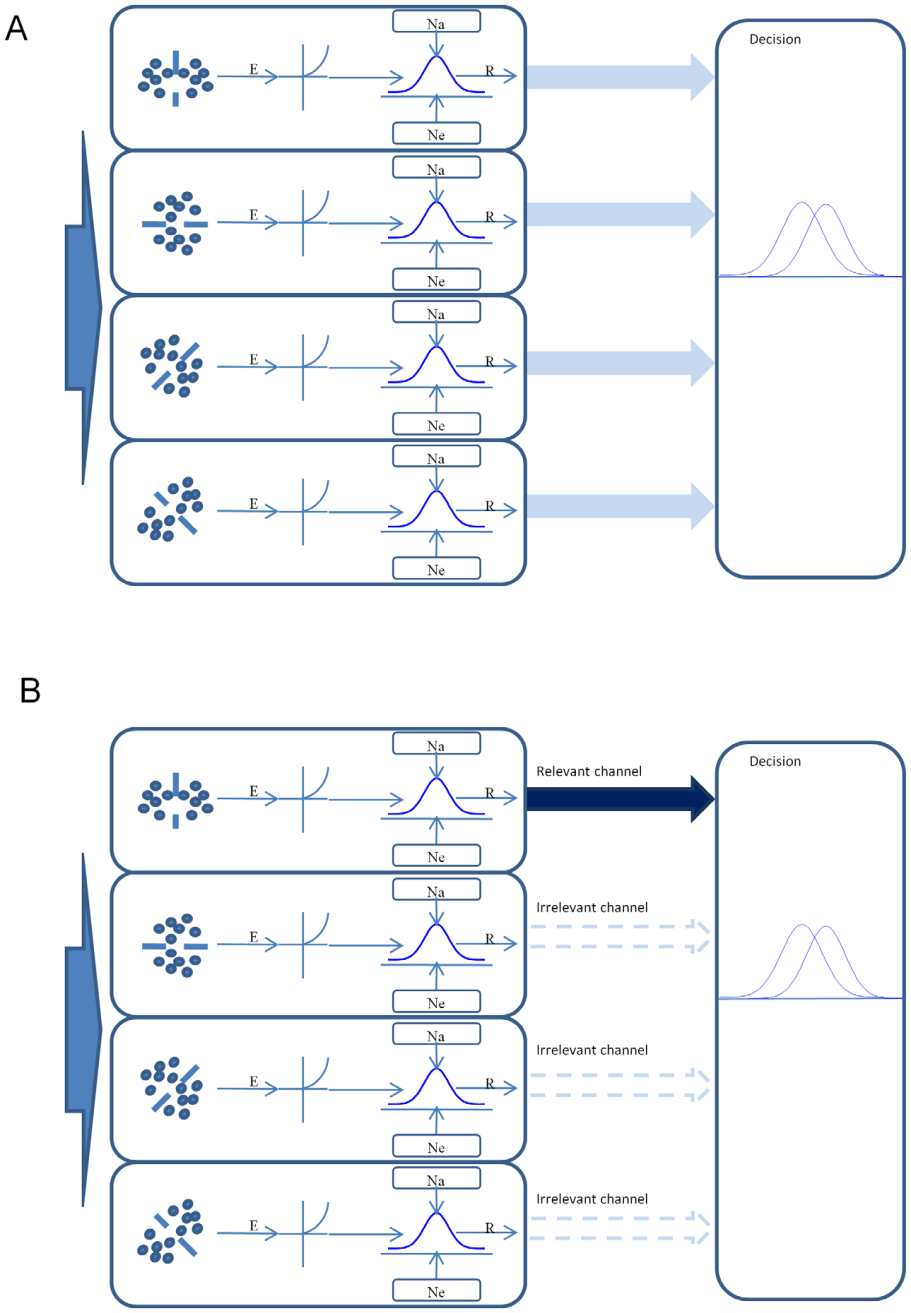
Diagram of the model. A. Without cues. B. With cues. See text for details.

Here, we present a model that can explain all aspects of our data. This model, in the framework of Signal Detection Theory [24], contains two stages: a perception stage and a decision stage (Figure 5). The perception stage concerns the noise-limited sensitivity of a visual mechanism to the stimuli limited by both internal and external noise, while the decision stage concerns the effect of uncertainty and cueing on the decision criterion.

#### Symmetry processing

The first step of the perception stage is a band of orientation-selective symmetry processors that are sensitive to symmetry in an image. Each processor is sensitive to the mirror symmetry about one axis. Note that these are not the traditional local filters but long-range pairs of local multiplicative contrast detectors that register a signal whenever there is a similar contrast at two locations in the field equidistant from a symmetry axis. The outputs of all such pairs of detectors relative to a given symmetry axis are linearly summed to form the symmetry signal relative to that location. It is important to emphasize that symmetry processing requires such axis selectivity, since any binary noise pattern has an infinite number of dot pairings at arbitrary locations and pairwise orientations. It is only when a number of them line up with respect to a particular symmetry axis or axes that we say that the pattern has symmetry.

The image in the target+masker trial can be considered to consist of two components: the symmetric target and the noise masker while the image in the control+masker trial can be considered to consist of just one component with a density that is the sum of the control and the masker.

For sparse binary random-dot patterns, such as those in our experiment, the output of the j-th processor to the i-th image component, E_j,i_, is
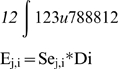
(2)where Se_j,i_ is the sensitivity, or gain factor, of the j-th processor to i-th image component, while D_i_ is the dot density of the i-th image component. Eq. (2) can be derived in many ways. For instance, one can simply calculate the correspondence of dots in the opposite regions of the image component about the symmetry axis, or reverse mapping [9], [17], [36] as 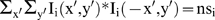
(3)where x' = x*cosθ+y*sinθ and y' = y*cosθ - x*sinθ with θ denoting the orientation of the symmetry axis and ns_i_ denoting the number of dots that have a corresponding dot in the other half of the image component i. For the same type of image, ns_i_ should be proportional to the number of dots in the image. That is, the covariance can be written as a_j,i_ * n_i_ where n is the number of dots in the i-th image component and a is a constant. The value of a_j,i_ depends on the type of the i-th image component and the property of the j-th processor. If the i-th image component is symmetrical about the axis to which the j-th processor is sensitive, a_j,i_ will be large; otherwise, a_j,I_ will be small. Scaling by the total number of possible pixels in an image, we then arrive at Eq. (2).

Eq. (2) should hold for spatial filtering approaches [20], [22], [37] as well. For the sparse random-dot patterns we used, each additional dot increases the contrast energy in the image by the same amount. Hence, the response of a linear filter should increase in proportion to the dot density. Thus, Eq. (2) should be reasonable way to describe the output of an orientation-specific symmetry processor.

#### Nonlinear response

The response of the perception stage of the model is the excitation of the j-th processor, E_j_, raised by a power p, in which E_j_ = Σ_i_ E_i,j_ is the sum of excitations produced by all image components, and is then divided by a divisive inhibition term I_j_ plus an additive constant z. That is,
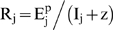
(4)where I_j_ is the summation of a non-linear combination of the excitations of all relevant mechanisms to mechanism j. This divisive inhibition term Ij can be represented as
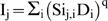
(5)where S_ij,i_ is a positive value serving as an inhibitory term.

#### Noise

The contribution of each channel to the visual performance is limited by the noise. There are two sources of noise in this model: the internal noise inherited in the system, and the external noise provide by the noise patterns. The variability of the internal noise, σ_a_^2^, is a constant for all processors in the model. The variability of the external noise, σ_e_^2^ is proportional to the square of the density of random-dot patterns, D_b_; that is, σ_e_^2^ = v * D_b_^2^, where v is a scalar constant. Pooled together, in each channel the standard deviation of the response distribution is 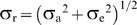
(6)


#### Decision stage

The output of the perception stage is sent to the decision stage. The decision stage monitors more channels than those that are relevant to the prescribed visual tasks [27]. Here, a relevant channel is the one whose symmetry selectivity matches that of the image. The performance of the system is limited not only by the noise in the relevant channels but also by that in the irrelevant channels. In our experiment, the task of the observer is to detect the symmetry in the image regardless the orientation of the symmetry axis. That is, the observer detects a symmetry pattern if the maximum response of all monitored channels to an image is greater than the response of a random-dot pattern by an amount that exceeds the level of noise in the system [24].

When there are m channels to be monitored, the maximum response of these channels can be described by a distribution whose mean approximates a fourth-power summation over these m channels [27], [38], [39], although the Gaussian distribution theory of Tyler & Chen [28] shows that the fourth power exponent is valid only under the restricted conditions of a particular attention model and a linear signal transducer. For the random-dot patterns, the mean of the response R'_b+c_ is
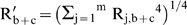
(7)


Here, we use the subscript b+c to emphasize that the noise pattern contained both the masking noise and a control pattern with same number of dots as the corresponding symmetry target. Suppose that there are n channels responding the symmetry image component in the stimuli when it is available. Then, the mean response in the decision stage becomes 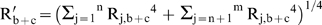
(7′)where the subscript t denotes the target or symmetry component. In our experiment, there were four possible symmetry axis orientations but only one was presented in the image. Thus, we assign m = 4 and n = 1 for the non-cued condition. In the cued condition, the observer needed only to monitor the relevant channel and thus m = n = 1.

The decision variable is the difference of the response to the image with the symmetry component and the response to the random-dot image of the same pattern divided by the standard deviation of the max distribution, σ_p_. That is,
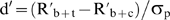
(8)


The threshold is defined when d' reaches unity. Note that the standard deviation of the max distribution of four independently and identically distributed samples is 0.71 times the standard deviation of the original distribution [35]. Thus, σ_p_ = σ_r_ for the cued conditions and σ_p_ = 0.71*σ_r_ for the non-cued conditions.

#### Model implementation and performance

In practice, if we use a typical value of 2 for the power for the divisive inhibition input q in Eq. (5) [40], [41], [42], we can combine the divisive inhibition terms and the noise terms and simplify the model by approximating the response of the individual channel in Eq. (4) by,
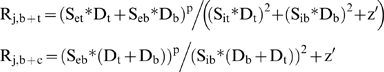
(4′)where D_t_ and D_b_ are the target and noise densities, respectively, and S_et_, S_eb_, S_it_, S_ib_, z' and p are the parameters in the model. Recall that noise patterns contain the same number of dots as the noise-plus-target (D_b_+D_t_) patterns. The decision variable of Eq. (8) thus becomes
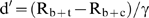
(8′)where γ = 1 for the cued condition and 0.71 for the non-cued condition.

Eqs. (4)′, (7) and (8)′ thus define the whole computation and all the parameters in the model. In general, the parameters in Eq. (4)′ were set the same for all conditions except as follows: we allowed the target-related sensitivity parameters S_et_ and S_it_ to change with axial salience as images with different salience were physically different. As discussed above, uncertainty reduction alone cannot explain the whole cueing effect. Other parameters also need to be adjusted to model the cueing effect. From Figure 3, we showed that the cueing effect was relatively constant for all masking densities. Hence, it is less likely that the cueing effect acted on the denominator of the response function, which would be noise-density dependent [43]. Instead, such an effect would be consistent with a change in the excitatory sensitivity to the target (S_et_ in Eq (4)′). Hence, in the model, we allowed S_et_ to be different for different cueing conditions. This arrangement is consistent with the signal enhancement theory of cueing effects [25], [44]. The parameter S_et_ for the high salience target in the cued condition was set to 1000 as an anchor point. Thus, in total, there were nine free parameters in the model for each observer.

**Figure 6:**
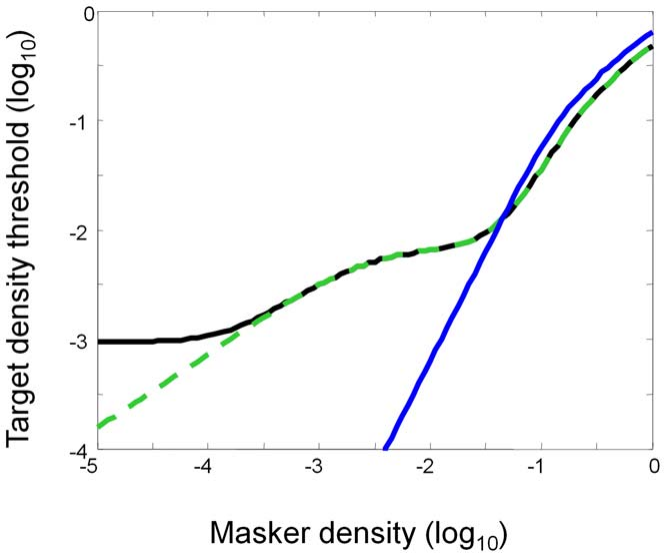
Examples of the model parametrization. The black curve has all three model components in the denominators, with parameters chosen to for a strong double-corner effect. Green curve: removing the internal noise reduces the threshold at low masker density. Blue curve: removing the divisive inhibition limits the masking effect at high masker density. (Removing the external noise results in a horizontal line, since it is the parameter of the x axis.)

Before describing the model fits, it is relevant to consider the inherent properties of the model. In particular, it has the property that the noise masking function can exhibit two “corners”, or locations where the slope of TvD function increases, instead of one as commonly seen in the discrimination functions in the contrast or luminance domain [25], [41], [45]. In the case of ***contrast*** discrimination functions, such corners reflect the transition between dominating terms in the denominator of the response function [43]: at low contrasts, it is the additive constant, z in Eq. (4), that dominates the denominator of the response function, while at high contrasts the divisive inhibition term or I in Eq. (4). In our model, there were three terms in the denominator of the response function from different sources: the “gain control” (or self inhibition) from the symmetry target, the external noise and the intrinsic noise or additive constant. The multiple corners in the fitted TvD functions reflect the transitions among these terms, as illustrated by the parametrized example in Figure 6.

The black curve contains all three components in the denominators of Eq (4)′. The parameters in this illustration were chosen to make both the two corners more pronounced. The green curve of Figure 6 shows the effect of removing the additive constant, i.e., z' in Eq (4)′; the threshold at low masker density is no longer limited by the intrinsic noise and is markedly reduced. At the high masker density, however, the TvD function is unaffected. On the other hand, if we remove the divisive inhibition by setting S_it_ in Eq. (4)′ to zero (blue curve in Figure 6), the TvD function is dominated by the external noise. Hence, the effect on masker-densitydependent threshold change is not pronounced until high masker density, illustrating that this part of the curve is dominated by the divisive inhibition term. (Removing the external noise is not shown because it is a degenerate case that results in a horizontal line, since it is the parameter of the x axis.)

#### Model fits

The model fits are shown as smooth curves in Figure 2. This model explains 97%–98% of the all variability in the thresholds across observers (112 free parameters). The root mean square error (RMSE) is between 0.07 to 0.10 log unit across observers, on par with the average standard error of measurement. Table 1 shows the fitted model parameters.

**Table 1:** Fitted model parameters.

		CC	LY	TR	HP
S_et_	cued, high axial salience	1000*	1000*	1000*	1000*
	cued, low axial salience	401	443	867	477
	non-cued, high axial salience	540	466	528	549
	non-cued, low axial salience	201	268	416	362
S_eb_		1.68	270	0.10	247
S_it_	high axial salience	890	850	400	1060
	low axial salience	60	750	260	780
S_ib_		1196	2007	7132	15219
z		0.15	0.03	3.54	2145
p		2.17	2.23	2.60	2.91
Other fixed parameters used in the model fits					
q		2*	2*	2*	2*
m	cued	1*	1*	1*	1*
	non- cue	4*	4*	4*	4*
n		1*	1*	1*	1*
γ	cued	1*	1*	1*	1*
	non-cued	0.71*	0.71*	0.71*	0.71*

*Fixed value, not a free parameter.

The ***excitatory sensitivity*** to the symmetric target was reduced by around 50% when prior knowledge of the symmetric axis orientation was not available to the observer. This sensitivity change is essential to explain the cueing effect. If we make the assumption that the cueing effect can be explained by the uncertainty reduction alone, the RMSE of the model fit increases 2–4 fold. This difference is significant even given the reduced number of free parameters in the uncertainty reduction account (F(2,15) = 13.28, p = 0.0004 for HP, F(2,18) = 4.61 to 43.89, p = 0.02 to 10^−7^ for other observers). Hence, our result provides strong evidence for the enhancement of symmetry sensitivity by prior knowledge about the symmetry axis.

The excitatory sensitivity to the low salience targets shows a 15–60% reduction compared with that to the high salience ones. This degree of reduction may be taken as an index of the relative contribution of the information at or near the symmetry axis. This result also suggests that 40–85% of symmetry sensitivity is from sources distant from the symmetry axis (by more than 0.58°). Notice that, since the distance between two neighboring apertures is 1.5° in our stimuli, such a contribution must be from a long-range interaction mechanism [1], [46]. The contribution needed for divisive inhibition from the low salience target is also smaller than that from the high salience target. Such reduced divisive inhibition balances out the reduced excitatory sensitivity and allows a relatively stable symmetry detection threshold across salience conditions. However, as the external noise level increases, the denominator of the response function (Eq. (4)′) is gradually dominated by the noise and the effect of the divisive inhibition diminishes. Hence, the difference in the excitatory sensitivity, and in turn, the symmetry detection threshold between two salience conditions, becomes more pronounced at high noise levels.

### Conclusion

The target threshold vs. mask density function for symmetry detection was flat at low mask density and increased with a slope of 0.75–0.8 beyond a critical density. The axis cueing reduced the target threshold 2–4-fold at all masker densities. On the other hand, axis salience, whether the paraxial dots were visible in the windows or not, had an effect only at high masker densities. These results are inconsistent with a signal-to-noise account of symmetry detection but can be explained by a multiple-channel model is which the response in each channel is limited by the nonlinear transform of early symmetry detectors combined with the sum of separate sources of external and intrinsic noise.

The combined design of the present study revealed that the near-axis region, which is often considered to be the sole determinant of symmetry detection, plays little role under noise-limited conditions, since masking it from view has only a small effect on detectability. Overall, the results are inconsistent with all published models of symmetry processing of which we are aware. The data require a more elaborated model of the form that we propose, consisting of a band of local-feature-selective symmetry processors configured as long-range pairs of local multiplicative contrast detectors that register similar contrasts at pairs of locations equidistant from a symmetry axis. The primary symmetry signal from all such pairs of detectors is linearly summed relative to a prescribed symmetry axis, subject to an inhibitory gain control based on the external noise level which is then sent to a decision stage that optimizes the response relative to the prior knowledge of the axis location. This model accounts for all the parametric variance in the data, including the minor individual differences among observers. We therefore regard the noise masking and axis salience properties as key variables in discriminating among symmetry models, and as providing strong evidence in favor of the current model structure for this form of mid-level processing for object recognition.
