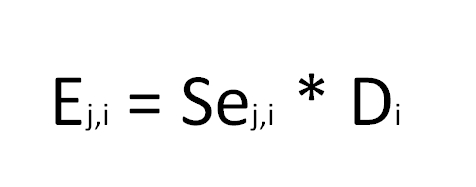# Correction: Symmetry: Modeling the Effects of Masking Noise, Axial Cueing and Salience

**DOI:** 10.1371/annotation/536a5de8-2f95-49e3-a683-af8b1e8207a8

**Published:** 2010-04-27

**Authors:** Chien-Chung Chen, Christopher W. Tyler

Equation 2 contains an error. The first line, which contains an integral sign, should not be included. Please view the correct equation here: